# Polymorphism Identification and Improved Genome Annotation of *Brassica rapa* Through Deep RNA Sequencing

**DOI:** 10.1534/g3.114.012526

**Published:** 2014-08-12

**Authors:** Upendra Kumar Devisetty, Michael F. Covington, An V. Tat, Saradadevi Lekkala, Julin N. Maloof

**Affiliations:** Department of Plant Biology, University of California, Davis, California 95616

**Keywords:** *Brassica rapa*, RNA-Seq, transcriptome, SNPs, genome annotation

## Abstract

The mapping and functional analysis of quantitative traits in *Brassica rapa* can be greatly improved with the availability of physically positioned, gene-based genetic markers and accurate genome annotation. In this study, deep transcriptome RNA sequencing (RNA-Seq) of *Brassica rapa* was undertaken with two objectives: SNP detection and improved transcriptome annotation. We performed SNP detection on two varieties that are parents of a mapping population to aid in development of a marker system for this population and subsequent development of high-resolution genetic map. An improved *Brassica rapa* transcriptome was constructed to detect novel transcripts and to improve the current genome annotation. This is useful for accurate mRNA abundance and detection of expression QTL (eQTLs) in mapping populations. Deep RNA-Seq of two *Brassica rapa* genotypes—R500 (var. trilocularis, Yellow Sarson) and IMB211 (a rapid cycling variety)—using eight different tissues (root, internode, leaf, petiole, apical meristem, floral meristem, silique, and seedling) grown across three different environments (growth chamber, greenhouse and field) and under two different treatments (simulated sun and simulated shade) generated 2.3 billion high-quality Illumina reads. A total of 330,995 SNPs were identified in transcribed regions between the two genotypes with an average frequency of one SNP in every 200 bases. The deep RNA-Seq reassembled *Brassica rapa* transcriptome identified 44,239 protein-coding genes. Compared with current gene models of *B. rapa*, we detected 3537 novel transcripts, 23,754 gene models had structural modifications, and 3655 annotated proteins changed. Gaps in the current genome assembly of *B. rapa* are highlighted by our identification of 780 unmapped transcripts. All the SNPs, annotations, and predicted transcripts can be viewed at http://phytonetworks.ucdavis.edu/.

High-density molecular genetic markers are a valuable resource for assessing genetic diversity both within and between species. They are useful for the construction of high-resolution genetic maps, for genotyping segregating populations, for marker-assisted breeding, and for map-based cloning (Edwards and Batley 2004). Next-generation sequencing (NGS) has significantly improved discovery and genotyping of such markers, especially single nucleotide polymorphisms (SNPs) (Shendure and Ji 2008). NGS SNP discovery can be focused on coding regions using RNA-Seq, reducing costs compared with whole-genome sequencing (Trick *et al.* 2009; Lai *et al.* 2012; Koenig *et al.* 2013). For the current study, we are particularly interested in detecting SNPs between two *B. rapa* varieties (hereafter referred to as genotypes), R500 (Yellow Sarson variety), and IMB211 (a rapid cycling variety) because these are the parents of a mapping population. The R500 genotype is a seed-oil cultivar grown in India for more than 3000 years (Prakash 1980). The IMB211 genotype is derived from Wisconsin Fast Plant population and therefore has a rapid generation time (Dorn and Mitchell-Olds 1991; Mitchell-Olds 1996). These genotypes have contrasting life-history strategies; compared with IMB211, R500 flowers late, reaches a large size at flowering, and accumulates more biomass (Edwards *et al.* 2009; Edwards and Weinig 2011). Previously, a *B. rapa* genetic linkage map was constructed using RFLP markers for this population (Iniguez-Luy *et al.* 2009). However, this linkage map is of low density and may not provide precise and complete information about the numbers and location of QTL. SNP based markers located in coding regions (cSNPs), such as those defined by RNA-Seq, can be useful for the development of high-resolution genetic map of *B. rapa*.

In addition to physically positioned gene-based molecular markers, accurate and comprehensive genome annotation (*e.g.*, gene models) is imperative for functional studies. Genome annotations initially relied primarily on *ab initio* gene predictions and alignment of reference transcripts of related species [GENSCAN (Burge and Karlin 1997), GlimmerHMM (Majoros *et al.* 2004), and Fgenesh (Salamov and Solovyev 2000)]. However, these strategies often have problems identifying short exons and predicting very long exons, identifying nontranslated exons, and predicting genes that encode noncoding RNAs accurately. Genome annotation based on gene prediction programs that rely on ESTs also have problems. For instance, it has been estimated that most EST+homology–based annotations miss 20% to 40% of transcripts that are novel transcripts or that are transcribed only under highly specific tissue or environmental or treatment conditions (Brent 2008). Currently, the annotation of protein-coding genes in the *Brassica rapa* (Chiifu) genome mainly relied on ESTs data from databases and *in silico* gene models (Wang *et al.* 2011). Updating and improving the current genome annotation of *B. rapa* is essential for better functional analysis studies and more accurate mRNA abundance estimates.

In this study, we describe our approaches for the generation of coding SNPs (cSNPs) and re-annotation of *B. rapa* genome using deep RNA-Seq. Using these approaches, we were able to identify 330,995 putative cSNPs between the two *B. rapa* genotypes. In addition, we were able to detect 3537 previously uncharacterized *B. rapa* transcripts and updated 23,754 gene models of *B. rapa* genome.

## Materials and Methods

### Plant materials and sample collection

Two *Brassica rapa* genotypes—R500 and IMB211—were grown in three different locations for tissue collection for RNA-Seq library preparation. For sample collections from the growth chamber, both genotypes were germinated on 1/2 MS (Sigma) plates kept in dark for 3 d with 10 to 15 seeds per plate. Then, the plates were exposed to continuous light and grown at 22° in a Conviron walk-in growth chamber. The 10-d-old seedlings were transplanted to 3-inch pots and were divided into two groups. One group (simulated sun group) continued to grow under the conditions as described above [high-red to far-red ratio (R/FR), 2.0], and the second group (simulated shade group) was grown under a mixture of cool white and far-red fluorescent lights (low R/FR, 0.2) in a complete randomized design. Both conditions had PAR ranging from 80 to 100 µE. Ambient light conditions were measured using *LI-COR* radiospectrophotemeter (Li-Cor). The following samples were collected from the growth chamber–grown sun and shade plants. Whole seedlings were collected 10 d after germination. Roots were collected from 10-d-old seedlings. Vegetative meristems were collected from plants when the third leaf reached 1 mm. The stem between the fourth and fifth leaves was collected for internode tissue, and inflorescence meristems were collected when these meristems were fully formed. Leaf samples were collected 28 d after germination, and siliques were collected when they were fully mature. For greenhouse sample collection, both genotypes were germinated and grown as indicated above, and 10 d after germination the seedlings were transferred to a greenhouse, transplanted to soil, and subjected to nondense and dense treatments. For the dense treatment, four plants surrounded each plant in a 6-inch square pot; for the nondense treatment, a single plant was grown per 6-inch pot. Internode, leaf, and silique samples were collected as indicated above. Petioles were collected from first, second, and third mature leaves. Only leaf and silique samples were collected from field-grown *B. rapa* genotypes. All the collected samples were immediately frozen in liquid nitrogen before storage at −80° until RNA extraction and library preparation. Samples used in this study are listed in Table 1.

**Table 1:** List of tissue samples collected from *B. rapa* genotypes R500 and IMB211 across growth chamber, greenhouse, and field conditions

Tissue	Location	Treatment	Genotype	No. of Replicates
GC pool				
Apical Meristem	GC	Shade	IMB211	2
	GC	Shade	R500	2
	GC	Sun	R500	1
Leaf	GC	Shade	IMB211	3
	GC	Sun	IMB211	3
	GC	Shade	R500	3
	GC	Sun	R500	3
Floral Meristem	GC	Shade	R500	2
Internode	GC	Shade	IMB211	2
	GC	Sun	IMB211	3
	GC	Shade	R500	3
	GC	Sun	R500	3
Seedling	GC	Shade	IMB211	3
	GC	Sun	IMB211	3
	GC	Shade	R500	3
	GC	Sun	R500	3
Silique	GC	Shade	IMB211	3
	GC	Sun	IMB211	3
	GC	Shade	R500	3
	GC	Sun	R500	3
Root	GC	Sun	IMB211	3
	GC	Shade	IMB211	3
	GC	Sun	R500	3
	GC	Shade	R500	3
	**Total**			**66**
GH pool				
Leaf	F		IMB211	3
	F		R500	3
	GH	DP	IMB211	3
	GH	NDP	IMB211	3
	GH	DP	R500	3
	GH	NDP	R500	3
Internode	GH	DP	IMB211	3
	GH	NDP	IMB211	3
	GH	DP	R500	3
	GH	NDP	R500	3
Petiole	GH	DP	IMB211	3
	GH	NDP	IMB211	3
	GH	DP	R500	3
	GH	NDP	R500	3
Silique	F		IMB211	3
	F		R500	3
	GH	DP	IMB211	3
	GH	NDP	IMB211	3
	GH	DP	R500	3
	GH	NDP	R500	3
	**Total**			**60**

GC, growth chamber; GH, greenhouse; F, field; DP, dense planting; NDP, nondense planting.

### Total RNA extraction, RNA-Seq library preparation, and sequencing

Total RNA was extracted from two to three biological replicates of different samples of *B. rapa* genotypes (Table 1) and purified using RNAeasy Plant Mini Kit (Qiagen). DNaseI (Qiagen) was used to remove any contaminating DNA according to the manufacturer’s instructions. The quality and quantity of the extracted RNA were initially assessed by NanoDrop ND 1000 (NanoDrop technologies). RNA-Seq libraries were prepared using Illumina’s TruSeq v1 RNA sample Preparation kit (RS-930-2002) with a Low-Throughput protocol following manufacturer’s instructions with the following modifications. All the reaction volumes were reduced to half to reduce costs. Custom paired-end barcoded adapters (Kumar *et al.* 2012) were used instead of Illumina’s RNA indexed adapters to multiplex the samples, 10 cycles of PCR enrichment was performed instead of 15 cycles to reduce amplification bias, and, finally, the libraries were constructed with an insert size of 300 to 350bp. The enriched libraries were then quantified on an Analyst Plate Reader (LJL Biosystems) using SYBR Green I reagent (Invitrogen). Once the concentration of libraries was determined, two pools were made to a final concentration of 20 nM, with one pool consisting of 66 samples collected from growth chamber and another pool consisting of 60 samples collected from greenhouse and field (Table 1). Each pool was sequenced on eight lanes (total 16 lanes) of an Illumina Genome Analyzer (GAIIx) as 100-bp paired end reads. The libraries that failed from both the pools were further sequenced on one extra lane (Table 2).

**Table 2:** Summary of RNA-Seq data obtained from *B. rapa* deep transcriptome using Illumina GAIIx sequencing

Pool Name/Run	Pool Number	No. of Tissues	Total No. of Reads	Fastq File Size (in GB)	Total No. of Reads After Quality Control	Average Read Length (bp)
s_6_1	1	66	218,642,024	58	162,403,886	100
s_7_1	2	60	193,176,106	50	148,346,164	100
GH_s_1	1	60	184,259,308	48	162,260,599	89
GH_s_2	1	60	181,519,358	48	160,143,988	89
GH_s_3	1	60	180,575,544	48	159,927,822	100
GC_s_1	2	66	218,352,856	56	183,452,891	100
GC_s_2	2	66	213,474,480	56	172,832,712	100
GC_s_3	2	66	96,917,806	26	75,331,287	100
GC_s_4	2	66	212,749,370	56	180,334,866	100
GC_s_5	2	66	223,341,414	60	144,237,478	100
GC_s_6	2	66	201,966,154	54	135,778,620	100
GC_s_7	2	66	201,305,348	54	142,418,270	100
GH_s_4	1	60	166,999,240	44	117,413,176	100
GH_s_5	1	60	213,801,168	58	136,508,912	100
GH_s_6	1	60	216,036,902	58	142,306,342	100
GH_s_7	1	60	203,973,230	54	136,831,614	100
GCGH_s_1	R	8	226,938,148	60	189,740,545	100
**Total**			**3,354,028,456**	**888**	**2,550,269,172**	**98.7**

Pool 1 includes all 66 different tissues collected from growth chamber. Pool 2 includes all 60 different tissues collected from greenhouse and field. Pool R includes all eight tissues that failed in pool 1 and pool 2.

### Pre-processing of Illumina RNA-Seq raw reads

The FastX-tool kit software (http://hannonlab.cshl.edu/fastx_toolkit/) and custom perl scripts were used to perform pre-processing of Illumina raw reads to ensure the good quality of sequencing reads for downstream analysis. The raw reads were either quality-filtered with fastq_quality_filter with parameters [q 20, p 85] or, in some cases, trimmed first using fastx_trimmer with parameters [f 1,3; l 78] and then quality-filtered with the same parameters as indicated above. Next, reads containing custom adapters were removed using a custom script. The reads were then sorted (de-multiplexed) by their custom barcode sequences using fastx_barcode_splitter with default parameters. The quality control of paired end reads resulted in some reads losing their partner reads. Further processing to extract properly paired reads and unpaired reads (orphan reads) was performed using a custom script. Only paired reads were kept for downstream analyses. Reads were checked for quality before and after quality control with FastQC quality assessment software (http://www.bioinformatics.babraham.ac.uk/projects/fastqc/).

### SNP detection

SNPs were obtained from the alignments of all available reads of each genotype separately (NCBI SRA accessions are listed in Supporting Information, Table S3) to *B. rapa* genome reference v1.2 data of Chiifu available from BRAD (http://brassicadb.org/brad/) using BWA v0.6.1-r104 (Li and Durbin 2009) with parameters [k 1, l 25, n 0.02, e 15, i 10]. All unmapped reads from BWA were mapped to putative splice junctions using TopHat v1.4.1 (Trapnell *et al.* 2009) with the following parameters [–segment-mismatches 1–max-multihits 1–segment-length 22–butterfly-search–max-intron-length 5000]. The resulting alignment files from Tophat and BWA were merged, and SAMtools v0.1.18 (Li *et al.* 2009) followed by Picard **(**http://picard.sourceforge.net/**)** were used to filter uniquely mapped reads and remove duplicated reads, respectively. Sequence polymorphisms between R500 and IMB211 were identified using a variant-detection, genotype-scoring, and visualization tool that we developed, SNPtools v0.1.5 (https://github.com/mfcovington/SNPtools/). R500 compared with IMB211 variants were deduced by comparing the lists of Chiifu-related variants. For example, an R500:Chiifu SNP would also be an R500:IMB211 SNP if, at the position in question, the IMB211 alignment to Chiifu has sufficient coverage and IMB211 matches the Chiifu reference. To refine the R500:IMB211 polymorphism list, we used the SNPtools noise-reduction feature. The initial set of R500:IMB211 polymorphisms was used by SNPtools to interrogate the R500 and IMB211 alignments at the position of each putative polymorphism. For each of these positions, the resulting genotype files indicate the number of reads matching each allele. For a true R500:IMB211 polymorphism, the majority of reads should match the appropriate allele for both alignments. Putative polymorphisms that went against this expectation were filtered out from the final list. The code that was used to perform each of these steps is available at https://github.com/MaloofLab/devisetty-g3-2014/.

### SNP annotation

The categorization of SNP effects was performed using SnpEff v3.0 (Cingolani *et al.* 2012). First, *B. rapa* genome annotation information in GFF3 format was retrieved from BRAD (*Brapa_gene*_v1.2.gff). This genome annotation provided predicted exon–intron gene structure. The default parameters of snpEff were used to perform the variant effect analysis. Both HTML and text output files were generated from snpEff and were used to perform the SNP annotation on the basis of their structural occurrence in the intergenic, intronic, and exonic regions. SNPs located in the exonic region were further categorized as CDS, 5′-region, and 3′-region. Depending on whether SNPs caused changes in the coding of an amino acid, SNPs in the CDS region of the protein-coding genes were annotated according to functional relevance as synonymous or nonsynonymous mutations.

### Experimental validation of SNPs

For validation of identified SNPs, 300-base-long sequence fragments (100 of them) containing a predicted SNP were excised from *B. rapa* genome using a custom script, and the Primer3Plus primer design tool (http://www.bioinformatics.nl/cgi-bin/primer3plus/primer3plus.cgi/) (Untergasser *et al.* 2007) was used to design primers. The designed primers are listed in Table S1. For verification of SNPs, genomic DNA was extracted from 7-d-old seedlings of the two *B. rapa* genotypes using a modified Dellaporta plant DNA extraction method (Dellaporta *et al.* 1983). Genomic DNA was diluted to 20 ng/μl and used as a template in a PCR final volume of 20 μl containing 1× Standard buffer (homemade), 200 μM of each dNTP (Promega), 0.25 μM of each primer, 0.1 μl of Taq DNA polymerase (homemade), and 2 μl of template DNA (20 ng/ul). The PCR amplification conditions include a 2-min denaturation step at 94° followed by 35 cycles of PCR (94° for 30 sec; 60° for 30 sec; 72° for 30 sec), with a final extension time of 7 min at 72°. The PCR products were purified using Axygen AxyPrep Mag PCR clean-up kit (MAG-PCR-CL-250) according to manufacturer’s protocol. The purified PCR products were then sequenced by Sanger DNA sequencing method with an ABI 3730 Capillary Electrophoresis Genetic Analyzer at UC Davis. Each of the sequences from both genotypes along with the Chiifu reference genome sequences were aligned and visually inspected by ebioX software (http://www.ebioinformatics.org/) to verify sequence polymorphisms. In addition to the estimation of true-positive and false-positive SNP rates, we also used these fragments to estimate the false-negative rate. To do this we identified all SNPs present in the Sanger reads in a 150-bp window surrounding the focal SNP (we only used 150 bp out of 300 bp because this region had the most consistently good sequence). For each SNP found in the Sanger reads, we then asked if there was coverage of this position in the RNA-Seq data, and if there was coverage whether we had detected the SNP. To further investigate apparent false-positive SNPs, we chose primer sets to amplify four apparent false-positive SNPs from recombinant inbred lines (RILs) predicted to be segregating for the SNPs in question. RNA-Seq libraries and genomic DNA were as used as template for RILs and parental genotypes, respectively. The amplification conditions and sequencing procedure are the same as above.

### *K*a/*K*s ratio computation

For a given transcribed region of the genome, *K*a denotes the average number of nonsynonymous substitutions per nonsynonymous site. Likewise, *K*s denotes the average number of synonymous substitutions per synonymous site. To estimate *K*a/*K*s ratio for the *B. rapa* genome, all the SNPs were first substituted into the Chiifu reference genome sequence using vcf2diploid v0.2.6 tool of AlleleSeq pipeline (http://info.gersteinlab.org/AlleleSeq). This generated separate genomes for R500 and IMB211. The corresponding CDS were then extracted using the gffread tool from Cufflinks package. The *K*a/*K*s ratio was computed using KaKs calculator (https://code.google.com/p/kaks-calculator/downloads/list). Tests were conducted to estimate the evolution of each codon using the “MLWL” method of codon substitution. To look for differences in *KaKs* ratios among chromosomes, we calculated the difference in median *KaKs* for each chromosome compared with the rest of the genome. We repeated this for 1000 permuted chromosomes. A chromosome was considered to have a significantly different *KaKs* ratio if the absolute value of the difference in its *KaKs* ratio (compared with the rest of the genome) exceeded the absolute value of the differences in 95% of the permuted datasets. In the first version of this test (to asses overall differences in *KaKs*), genes were randomly assigned to the permuted chromosomes (keeping the number of genes the same as in the original data set). To test for the contribution of differential sub-genome composition per chromosome, we assigned genes to chromosomes in a way that preserved the proportion of LF, MF1, and MF2 genes on each chromosome.

### *De novo* transcriptome assembly methods

Post-processed paired end reads of *Brassica rapa* R500 genotype from 10 Illumina GAIIx lanes (NCBI SRA accessions SRR1227842, SRR1228204, SRR1228205, SRR1228206, SRR1228207, SRR1228208, SRR1228209, SRR1228210, SRR1228211, SRR1228212) (Table S3) were pooled together and assembled *de novo* using Velvet v1.2.07 (Zerbino and Birney 2008) followed by Oases v0.2.08 (Schulz *et al.* 2012) on XSEDE Lonestar computer cluster (https://www.xsede.org/tacc-lonestar) and Trinity r2012-06-08 (Garber *et al.* 2011) on XSEDE Blacklight computer clusters (http://www.psc.edu/index.php/computing-resources/blacklight). Because of the highly variable nature of transcriptome coverage (Surget-Groba and Montoya-Burgos 2010), we chose seven different k-mer sizes (31, 35, 41, 45, 51, 55, and 61) to increase the chances of transcript assembly using Velvet-Oases. In addition, we also tested two merged assemblies with k-mer sizes 27 and 55 using Oases merge. We analyzed various assembly parameters such as total number of transcripts, N50 (N50 is a weighted median statistic indicating that 50% of the entire assembly is contained in contigs equal to or larger than this value), longest transcript length, and average transcript length as a function of k-mer length. To produce a nonredundant set of Oases mRNA sequences rather than include alternative splicing, we used a custom python script (https://code.google.com/p/oases-to-csv/downloads/list) to choose a single representative transcript based on coverage and sequence length. Next, to assess the quality and completeness of the assembly, we then compared the current Velvet-Oases assembly with *B. rapa* genome (Brapa_sequence_v1.2.fa), *B. rapa* CDS (*Brassica_rapa*_v1.2.cds), and NCBI nr database. Finally, the blast output was fed into custom Perl script to remove any transcripts that hit multiple chromosomes at different locations (chimeras) to generate novel Velvet-Oases transcripts. Trinity was run on the paired-end reads with one k-mer size of 25 using the following parameters: minimum contig length = 200; paired fragment length = 500; CPUs = 16; and a butterfly HeapSpace = 10G. For each of the assemblies, first the splicing transcripts were removed by blasting them to the publically available plant refseq database (ftp://ftp.ncbi.nlm.nih.gov/refseq/release/plant/plant.1.protein.faa.gz) (Pruitt *et al.* 2007) and NCBI nr database, followed by chimera removal to generate novel Trinity transcripts.

### Reference-based transcriptome assembly methods

For reference-based transcriptome assembly, high-quality reads from three Illumina GAIIx lanes (NCBI SRA accessions SRR1227842, SRR1228204 and SRR1238058) (Table S3) were first aligned to *B. rapa* genome data using TopHat v1.4.1 with default parameters. The aligned reads from TopHat were then processed by Cufflinks v2.0.2 to assemble into transcripts. Cufflinks may or may not use existing gene annotation during assembly of transcripts, but rather it constructs a minimum set of transcripts that best describe the reads in the data. The current assembly was performed both with and without the help of reference annotation to capture both novel and native transcripts. We used default parameters for Cufflinks. Once all the transcripts were assembled with Cufflinks, the output GTF file (transcripts.gtf) was then fed to Cuffcompare v2.0.2 (Trapnell *et al.* 2010) along with the reference GTF annotation file (Brapa_gene_v1.2.gff) downloaded from BRAD. This classified each transcript into different class codes. The classification basically describes the nature of the match to the reference gene annotation (http://cufflinks.cbcb.umd.edu/manual.html#tmap). For current assembly purposes, only class code “u” transcripts (unknown intergenic transcripts according to Cufflinks) were considered to generate novel TopHat transcripts using gffread utility in Cufflinks package.

### Annotation of novel transcripts

To lower the redundancy among novel transcripts resulting from Velvet-Oases, Trinity, and TopHat-Cufflink pipelines, they were first pooled and CAP3 (Huang and Madan 1999) was used with default parameters. The resulting contigs and singletons from CAP3 were concatenated and used for downstream analysis. For all transcripts, the open reading frames most likely to encode proteins were identified using the transdecoder package (http://transdecoder.sourceforge.net/) with default parameters except for minimum protein length [(m) = 50]. For multiple ORFs from the same transcript, we defined the primary transcript as the one with the maximum number of significant hits to NCBI nr database. To generate annotation, the ORF filtered transcripts were first mapped against *B. rapa* genome sequence with default parameters using Burrows-Wheeler Aligner (BWASW) (Li and Durbin 2009). To select only those unmapped transcripts that are likely to be real, they were queried against the NCBI nr database using BLASTX with an e-value threshold of 1e−06. Functional annotation of the unmapped transcripts was performed using Blast2GO annotation tool. The mapped transcripts from BWASW output were next converted to a bed file using bamtobed utility of BED Tools (http://bedtools.readthedocs.org/en/latest/). A custom script was next used to join the exons for each transcript in the bed file. A further filtering step was included to eliminate transcripts that are more than 10 kb because these most likely represent problems with *B. rapa* assembly. In cases when there were more than one isoform per transcript, the selection of the best isoform was based on maximum length of the transcript. Finally, a fasta file corresponding to the annotation file was generated using the getfasta utility from BED Tools.

### PASA annotation

The use of PASA (http://pasa.sourceforge.net/) (Haas *et al.* 2003) to assemble full-length transcripts based on RNA-Seq data has previously been described (Rhind *et al.* 2011). For the current analysis, PASA was used to update the existing genome annotation using evidence from *de novo* RNA-Seq assembly and reference-based RNA-Seq assembly. PASA updates pre-existing protein-coding gene annotations to incorporate the PASA alignment evidence, correcting exon boundaries, adding UTRs, and models for alternative splicing based on the PASA alignment assemblies generated. Default parameters were used and a total of three rounds of PASA annotation were performed. RSEM (http://trinityrnaseq.sourceforge.net/analysis/abundance_estimation.html) was used to further filter out lowly expressed alternatively spliced transcripts. Finally, a fasta file corresponding to PASA updated annotation was generated using getfasta utility from BED Tools.

### Semi-quantitative RT-PCR validation

For RT-PCR, total RNA from leaves, internodes, siliques, seedlings, and roots was extracted from *B. rapa* parental genotype R500 and treated with DNase I. Approximately 1 ug of purified total RNA from each sample was used for first-strand cDNA using oligo-dT_(18)_ primer (Sigma) and SuperScriptIII reverse-transcriptase (Invitrogen) according to manufacturer’s instructions. The cDNA was diluted 1 to 50 and equal quantities of first-strand cDNA were used as a template. A total of 70 transcripts (20 for each of Velvet-Oases, Trinity, TopHat-Cufflinks “u” transcripts and 10 for TopHat-Cufflinks “o” transcripts) were randomly selected and primer sets were designed to amplify 300-bp to 400-bp fragment using Primer3Plus tool. For TopHat-Cufflinks “o” transcripts, primers were designed in such a way that one of the primers from each set would anneal to the predicted novel exon. The actin gene (JN120480) was used as internal control. Primers used for RT-PCR validation are given in Table S2. RT-PCR amplification of template RNA from different samples was performed using ExTaq kit (TaKaRa) using manufacturer’s protocol. The PCR amplification conditions include a 98° hold for 2 min, followed by a 30 cycles at 98° for 30 sec, 60° for 30 sec, 72° for 30 sec, and a final extension at 72° for 7 min. Semi-quantitative analysis of the RT-PCR amplified products was performed by agarose gel electrophoresis.

### *In silico* RNA-Seq coverage and genome annotation validation

For *in silico* RNA-Seq coverage and genome annotation validation, Chiifu public RNA-Seq reads (SRR643621-SRR643628) were first mapped to *B. rapa* genome reference v1.2 using BWA v0.6.1-r104 with default parameters. All unmapped reads from BWA were next mapped to putative splice junctions using TopHat v1.4.1 with the following parameters (–segment-mismatches 1 –max-multihits 1 –segment-length 22 –butterfly-search–max-intron-length 5000). The resulting alignment files from BWA and Tophat were merged into a single alignment file. Finally, the merged bam files of R500 and Chiifu were loaded onto IGV (Thorvaldsdottir *et al.* 2013) along with original and updated genome annotation.

### Gene ontology (GO) annotation and functional classification

BLAST (Altschul *et al.* 1990) searches of the gene models were performed against the nonredundant (Nr) protein database at NCBI using a blast cut-off of 1e−03. All BLAST results were saved as XML. Blast2GO v2.5.0 (http://www.blast2go.org/) (Conesa *et al.* 2005) was used to assign gene ontology (GO) IDs to the gene models based on the BLASTX output. After blast, GO annotation was performed using an e-value cut-off of 1e−03, an annotation score cut-off of 45, and a GO weight of 5. After obtaining GO annotation for every unigene, WEGO software (http://wego.genomics.org.cn/cgi-bin/wego/index.pl/) (Ye *et al.* 2006) was then used to simplify the output for producing combined graphs for molecular function, cellular process, and biological process.

### Pathway mapping using KEGG

To determine gene ortholog assignment and pathway mapping of transcripts, Kyoto Encyclopedia Genes and Genomes (KEGG) mapping was used. The transcripts were initially mapped to KEGG metabolic pathway database by submitting the sequences to the Kyoto Encyclopedia of Genes and Genomes automatic annotation server (KAAS) (http://www.genome.jp/tools/kaas/) and the single-directional best hit (SBH) method was selected. KAAS annotates every submitted sequence with KEGG ontology (KO) identifiers, which represents an orthology group of genes directly linked to an object in the KEGG pathway and thus incorporates different types of relationships that exist in biological systems.

### *Brassica rapa* UCSC genome browser

A customized UCSC Genome Browser of *Brassica rapa* (http://phytonetworks.ucdavis.edu/) has been set-up as a community resource that provides an integrated display of annotation data (*B. rapa* novel transcripts annotation track, *B. rapa* existing annotation track, *B. rapa* updated annotation track), data containing our alignment results of *B. rapa* genotypes R500 and IMB211 with respect to *B. rapa* genome data v1.2 (R500 *vs.* IMB211, R500 *vs.* Chiifu, and IMB211 *vs.* Chiifu SNP tracks). The VCF files (File S1, File S2, File S3) for the SNP tracks were generated using a Perl script (parental-vcf-writer.pl) from https://github.com/mfcovington/vcf-generator. This script depends on the V*cf*.pm Perl module from VCFtools (Danecek *et al.* 2011).

### Data deposition

The reads were submitted to the NCBI sequence read archive (SRA) (http://www.ncbi.nlm.nih.gov/Traces/sra/sra.cgi/) with accession numbers listed in Table S3 and all the transcript-derived contigs have been submitted to the NCBI Transcriptome Shotgun Assembly (TSA) (https://submit.ncbi.nlm.nih.gov/subs/tsa/). This TSA project has been deposited at DDBJ/EMBL/GenBank under the accession number GBDX00000000. The version described in this article is the first version, GBDX01000000. Because TSA allows submission of fasta sequences only greater than 200 bp, the cDNA of all annotated transcripts of *B. rapa* were saved in File S4.

## Results and Discussion

### Overview of deep RNA-Seq data

To identify a large number of SNPs and to re-annotate the *B. rapa* genome, we performed deep RNA sequencing of two *Brassica rapa* genotypes using several tissues, environmental conditions, and treatments on multiple sequencing lanes (see *Materials and Methods*). Using Illumina sequencing, we generated 3.35 billion reads with an average read length of 98 bp encompassing approximately 880 GB of data in fastq format. From these 3.35 billion reads, we obtained 2.54 billion quality-filtered and trimmed reads after removal of low-quality reads, adapter reads, and primer reads (see *Materials and Methods*). The final sequencing results are summarized in Table 2. It is expected that the current data would have enough coverage to detect SNPs in expressed regions between the two genotypes, to detect rare novel transcripts, and to allow re-annotation of the *Brassica rapa* genome coding genes.

### SNP detection in *Brassica rapa* using deep RNA-Seq

For expressed genes, the use of RNA-Seq data for SNP detection can be advantageous because it enriches for expressed regions of the genome. Thus, in addition to providing genotyping SNPs, RNA-Seq–based SNP discovery enriches detection of functionally important SNPs. In the current study, all the reads corresponding to each genotype were pooled separately and used for SNP detection. The final number of reads for *B. rapa* genotypes (R500 and IMB211) are 1.26 and 1.08 billion reads, respectively. A total of 330,995 putative SNPs (in 18,143 BRAD gene models) were identified from *B. rapa* transcribed regions. Of these, 639,788 (in 24,966 BRAD gene models) and 595,619 (in 24,704 BRAD gene models) were between R500 and Chiifu and IMB211 and Chiifu, respectively (Table 3).

**Table 3:** Summary of total number of SNPs detected and annotated to different regions of the genome between Chiifu and two genotypes of *B. rapa*

	Total No. of Annotated SNPs	SNP Rate	Total Number of Exonic SNPs	Total No. of Intronic SNPs	Total No. of Intergenic SNPs	Total No. of Nonsynonymous Coding SNPs
R500 *vs.* IMB211	330,995	0.50	202,295	48,210	80,833	66,327
R500 *vs.* Chiifu	639,788	0.83	358,391	124,429	157,726	119,222
IMB211 *vs.* Chiifu	595,619	0.81	338,749	110,437	147,212	111,719

### Characterization of detected SNPs

**Figure 1:**
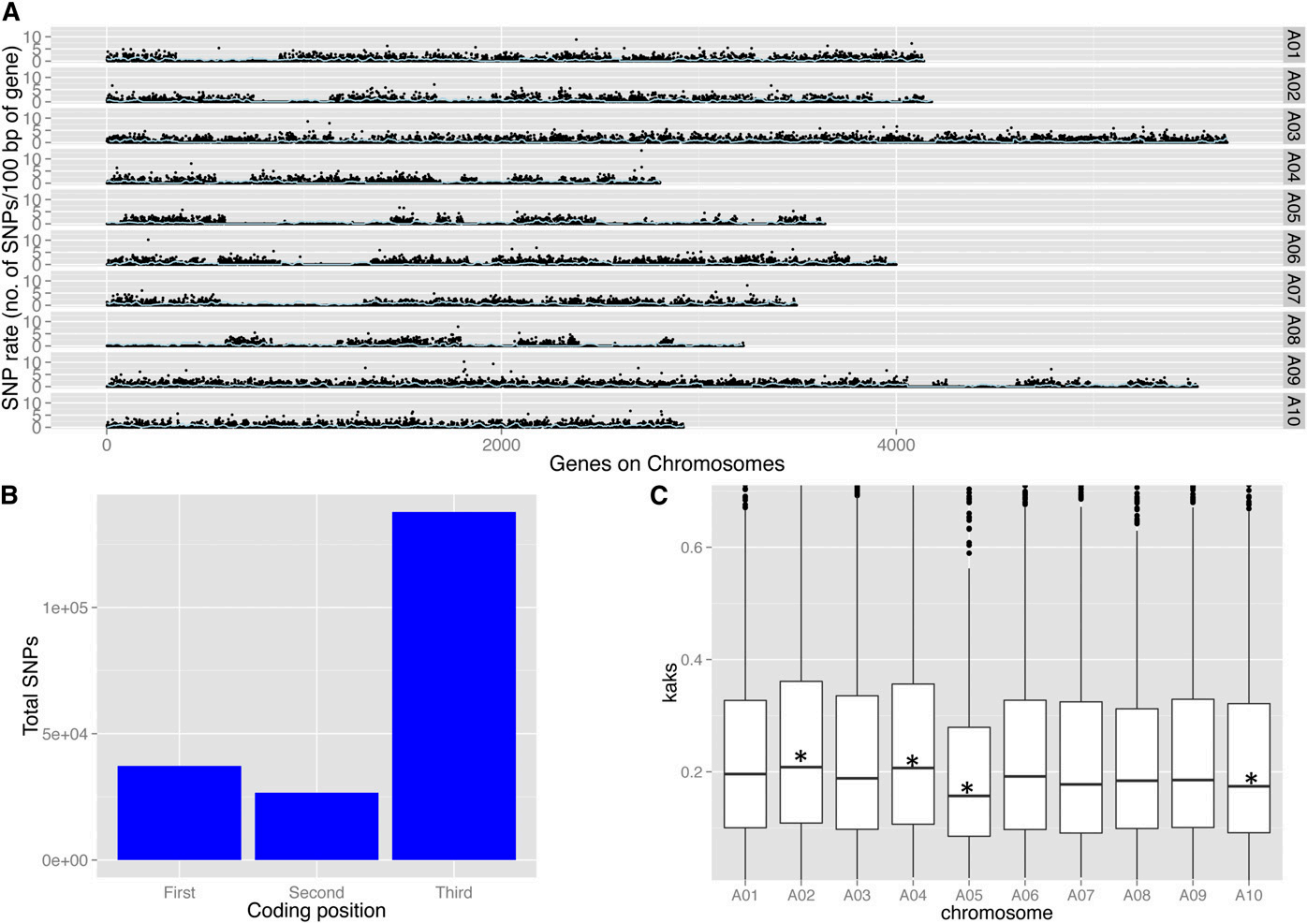
SNP annotation of *B. rapa* using snpeff. (A) SNP rate (total number of SNPs/100 bp of gene) across 10 chromosomes. Blue line indicates rolling mean across 25 genes. (B) The distribution of SNPs at different codon positions. (C) *K*aKs box plot of all chromosomes. Asterisks indicate significance at *P* ≤ 0.05 (permutation testing).

The distribution and frequency are important considerations of using SNPs as genetic markers. The current study found the SNP rate of uniquely mapped gene models showed a nonuniform distribution across the genome (Figure 1A). This observation could reflect low sequence coverage or segments more closely related because of the breeding history of these two genotypes or the presence of detected and undetected ancestral centromeres in the *B. rapa* genome (Cheng *et al.* 2013). Among regions with sufficient coverage for SNP detections (coverage of four or more reads, 66 to 77 million bp), the SNP rate was 1 SNP per 200 bp for R500 compared with IMB211, 1 SNP/120 bp for R500 compared with Chiifu, and 1 SNP/123 bp for IMB211 compared with Chiifu. In plants, SNP frequencies vary widely; for example, 1 SNP/124 bp in coding regions among 36 inbred lines of maize (Ching *et al.* 2002), 1 SNP/72 bp in expressed genes among 13 lines of sugar beet (Schneider *et al.* 2007), and 1 SNP/2.1 kb to 1 SNP/1.2 kb between two cultivars of *B. napus*, Tapidor, and Ningyou 7 (Trick *et al.* 2009). The SNP frequency observed in *Brassica rapa* coding regions is therefore within the range of those reported for other plant species. Out of 330,995 putative SNPs that were detected between R500 and IMB211, 202,295 (61%) were annotated in coding regions of *B. rapa* genome. Of these, 66,327 were nonsynonymous and 135,305 were synonymous changes. As expected, most SNPs occurred in the third nucleotide position of the codon unit (Figure 1B), suggesting that our SNP calling procedures are working correctly. A comparison of the ratio of *K*a to *K*s for all 10 chromosomes indicated that chromosomes two and four had significantly higher *KaKs* ratios than the rest of the genome, whereas chromosomes five and 10 had significantly lower ratios (Figure 1C). We hypothesized that this may be because of different proportions of the three sub-genomes of *B. rapa* because the nonsynonymous substitution rates are known to vary among the sub-genomes (Cheng *et al.* 2012). To test this, we calculated the *K*a*K*s for the three sub-genomes (see *Materials and Methods*) and found that the ratio of *K*a*K*s of LF (least fragmented) is significantly lower than MF1 and MF2 (more fragmented), similar to previous observations (Cheng *et al.* 2012). We next used a permutation-based approach to determine if the *KaKs* differences among chromosomes could be explained by sub-genome composition of each chromosome. We generated 1000 permuted genomes where, for each permutation, the proportion of LF, MF1, and MF2 genes on each chromosome matched the original dataset. We then asked how often the *KaKs* differences of the original chromosomes exceed those in the permuted dataset. The observed increased *KaKs* for chromosome two exceeded that in the 91% of the permuted datasets, suggesting that sub-genome composition may not entirely explain the high *KaKs* for this chromosome. In contrast, chromosome four exceeded the permuted *KaKs* differences only 46% of the time, indicating that its high *KaKs* rate can be explained by its sub-genome composition. Chromosome five had an unusually low *KaKs* rate that was lower than 99% of the permuted datasets, strongly suggesting that its low *KaKs* rate is driven by something other than sub-genome fractionation. Finally, the low *KaKs* of chromosome 10 was lower than 84% of the permuted datasets. In summary, sub-genome distribution can explain some, but not all, of the chromosomal variance in *KaKs*.

### Validation of putative SNPs

Sources of errors within NGS technologies could propagate to SNP detection and can ultimately result in poorly resolved genotypes (Depristo *et al.* 2011). To evaluate the performance of our SNP detection pipeline, primers were designed to validate putative SNPs detected between R500 and IMB211 (see *Materials and Methods*). We found that there were, in total, 202 SNPs (including the 100 SNPs we originally chose to validate) detected by RNA-Seq across these regions. Of these 202, there was no Sanger data available for 32 (failed PCR or sequencing), leaving 170 where we had both RNA-Seq and Sanger data. Among these 170, 158 were confirmed by Sanger sequencing. Considering only those positions with RNA-Seq and Sanger data, our validation rate was 93% (false-positive rate of 7%). Considering all 202 SNPs detected by RNA-Seq the validation rate was 78%, the false-positive rate was 6%, and 16% are unknown due to no Sanger data.

To investigate the basis of the 12 false positives, we re-examined the RNA-Seq data. At these apparent false-positive positions, the RNA-Seq data overwhelmingly support the presence of the SNPs: an average of 99.2% of the reads matched the SNP called with an average coverage of 368 and 263 reads for R500 and IMB211, respectively. We hypothesized that the difference between Sanger and RNA-Seq could be due to polymorphisms still segregating in the parental stocks. To test this idea, we used Sanger sequencing to assay four apparent false positives in RILs derived from these parents (see *Materials and Methods*). All four of the apparent false positive SNPs were detected in the RILs. Based on this result, we conclude that the apparent false-positive SNPs are generally not due to errors in our SNP detection pipeline but because the parental strains are not fully homogeneous (*i.e.*, there are some polymorphisms between the IMB211 used for our RNA-Seq and for Sanger validation).

To determine the false-negative rate, we identified all SNPs present in a 150-bp window surrounding the focal SNP in each Sanger reaction (193 SNPs total); 158 of these were called as SNPs from our RNA-Seq data. Of the remaining 35 where no SNP was detected by RNA-Seq, 29 were in regions with RNA-Seq coverage below the threshold required by our SNP detection pipeline (four reads in each genotype). This is probably because the Sanger sequencing was performed on genomic DNA and includes intronic and intergenic regions. The remaining six were not called as SNPs by our pipeline and represent false negatives (false-negative rate of 4% in regions with good RNA-Seq coverage).

One of the objectives of this study is the development of a marker system involving the genotyping of *B. rapa* genotypes by deep RNA-Seq for use in construction of recombination maps. Because of high validation rate of this dataset, the detected SNPs can now be used as markers to genotype the RIL mapping population generated from these two parents (Iniguez-Luy *et al.* 2009).

### R500 transcriptome assembly and re-annotation of *B. rapa* genome

**Figure 2:**
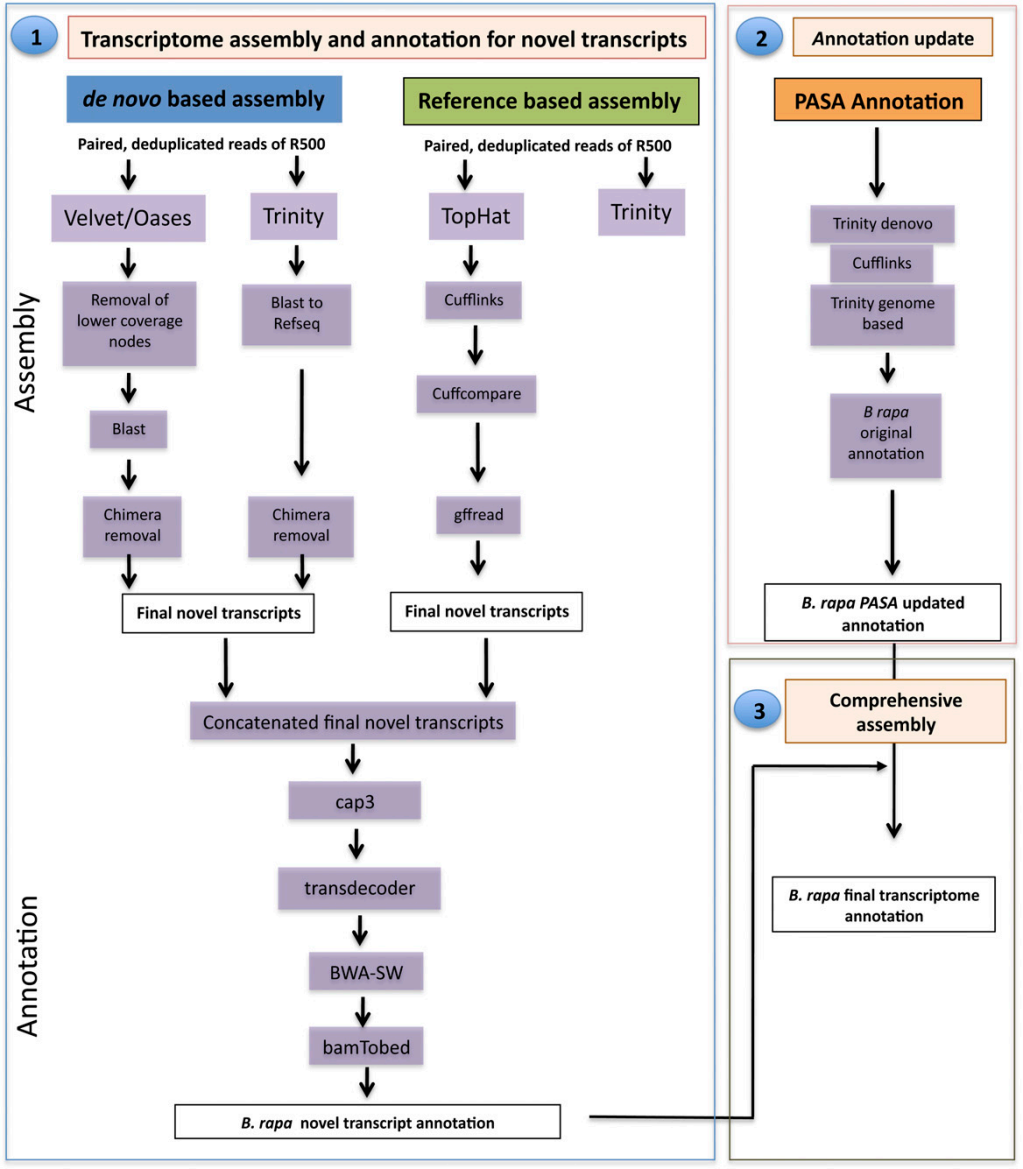
Pipeline illustrating the overall transcriptome assembly and annotation of *Brassica rapa* genotype R500.

The pipeline for R500 transcriptome assembly and re-annotation of *B. rapa* genome involved three steps. First, transcriptome sequence libraries were assembled and novel transcripts were detected and annotated. Second, the current genome annotation of *B. rapa* was updated and annotated. Third, both the novel annotated transcripts and updated annotated transcripts were combined to generate a final improved transcriptome assembly and genome annotation of *B. rapa* (Figure 2). For assembling and re-annotation purposes, we used reads from one genotype only (R500) to avoid mis-assemblies that could be caused by the approximately 330,000 SNPs existing between the two genotypes. In addition, we only used a subset of total reads for R500 to reduce computational requirements.

### Transcriptome assembly and annotation of novel transcripts

Currently, there are two basic approaches for transcriptome assembly using RNA-Seq technologies: *de novo*, which are sequenced reads that are directly assembled into transcripts without the need of reference, and reference-based, which are transcripts that are assembled by first mapping to a reference genome (Haas and Zody 2010). Both methods have advantages and disadvantages. For example, reference-based methods are computationally less demanding, tolerate sequencing errors, and detect repeats through alignment; however, these methods are dependent on a reference genome, assume that transcripts are collinear with the genome, and mismatched genome alignment or genome assembly errors lead to errors in transcriptome prediction. In contrast, *de novo* methods are not dependent on a reference genome and therefore can define transcripts not present in the reference as well as defining noncollinear transcripts and *trans*-spliced transcripts that result from chromosomal rearrangements. However, they will perform poorly on low-expressed genes and can produce chimeras and misassemblies due to repeats. In this study we used a hybrid approach that combines the novel transcripts from both *de novo*–based and reference-based strategies to ultimately incorporate these data into an updated annotation of *B. rapa*.

### Overview of assemblers used in this study

Velvet-Oases and Trinity were selected to *de novo* assemble the sequence reads. Although both these assemblers use similar de Bruijin graph algorithms, they differ in how they handle sequencing errors, resolve ambiguities, and utilize read pair information. For reference-based transcriptome assembly, we chose TopHat-Cufflinks assembly method.

### *de novo* assembly pipeline to detect novel transcripts

Because all *de novo* assemblers are computationally intensive, we used 396,946,216 high-quality reads from 10 lanes for *de novo* assembly using Velvet-Oases and Trinity (see *Materials and Methods*). The result from the Velvet-Oases pipeline indicated that in comparison with individual k-mer assemblies, the merged assembly with k-mer size of 55 yielded the best assembly because it had the maximum number of transcripts (601,915), largest average transcript size (1553 bp), largest transcript length (26,060 bp), and highest N50 length (2218 bp) (Table 4). A custom python script was then used to choose a single representative transcript for each gene based on coverage and sequence length (see *Materials and Methods*). This procedure yielded 43,816 best Velvet-Oases transcripts from 601,915 initial transcripts.

**Table 4:** Summary statistics for individual and merged Velvet-Oases assemblies

k-mers	No. of Transcripts	Total Bases (bp)	Average Transcript Length (bp)	Maximum Transcript Length (bp)	Minimum Transcript Length (bp)	N50	N90	No. of Transcripts in N50
31	227,834	233,436,468	1024	23,632	100	1959	489	36,982
35	210,673	231,120,029	1097	25,926	100	1987	551	36,312
41	182,288	222,326,942	1219	22,553	100	2022	640	34,524
45	169,361	213,517,894	1260	22,480	100	1989	665	33,885
51	151,970	196,156,152	1290	23,619	100	1898	672	32,425
55	140,093	191,427,390	1366	23,673	100	1976	751	30,947
61	123,880	167,489,435	1352	16,491	100	1908	734	28,093
Merged_27	577,900	895,807,087	1550	26,098	100	2214	833	128,986
Merged_55	601,915	935,108,204	1553	26,060	100	2218	855	134,411

To supplement the Velvet-Oases assembly, we used another *de novo* assembler, Trinity, which was shown to recover more full-length transcripts across a range of sensitivity levels similar to genome alignment methods (Grabherr *et al.* 2011). For Trinity, we used the same input reads as for Velvet-Oases. A total of 158,863 transcripts were assembled from Trinity; 61,438 (38.6%) of the assembled transcripts were more than 1000 bp long and 5107 (32.1%) were more than 4000 bp long. The mean transcript length, maximum transcript length, and N50 size of Trinity transcripts were 1112, 22,887, and 1863, respectively. To assess the Trinity assembly, bowtie was used to map the reads back to the Trinity assembly and 97% of the reads mapped to the assembly. To identify a core/reference set of transcripts from the 158,863 Trinity transcripts, we performed BLASTX of the Trinity transcripts against Plant RefSeq database and retained the 98,883 with homology to known plant proteins. The transcripts were then subjected to duplicate removal using a custom awk script, yielding 39,084 transcripts (see *Materials and Methods*).

After initial assembly with Velvet-Oases and Trinity, to extract novel transcripts not present in the existing *B. rapa* genome annotation, the transcripts were first blasted against the *B. rapa* CDS, downloaded from BRAD, using megablast (percent identity of ≥95 and e-value filter of 1e−06). To further determine if the extracted novel transcripts were *bona fide B. rapa* transcripts, these were blasted against the *B. rapa* genome using megablast (with the same parameters as above). Those transcripts that had genome matches were then subjected to chimera removal using a custom script. The Velvet-Oases transcripts that did not have a *B. rapa* genome match (due to either gaps or inaccuracies in the genome assembly or due to contamination) were blasted against the nr database of NCBI (e-value cut-off of 1e−06) and only those that have at least one hit on the nr database were kept. Because Trinity transcripts were already derived from blasting to NCBI Plant RefSeq, no further blasting was performed. Finally, the genome-matched novel transcripts (after removing chimeras) and nongenome-matched novel transcripts (after blasting to nr database) were merged separately for Velvet-Oases and Trinity to obtain assembly specific novel transcripts. The full results for each of the above steps are given in Table 5.

**Table 5:** Downstream processing of Velvet-Oases and Trinity transcripts after initial assembly

	Velvet-Oases	Trinity
a) Initial assembly transcripts	43,816	39,084
b) No. of novel transcripts remained after removing blast hits to *B. rapa* CDS	14,540	5464
c) No. of novel transcripts from (b) that have *B. rapa* genome blast hits	11,182	3789
d) Number of novel transcripts from (c) remaining after chimera removal	9448	2377
e) Number of novel transcripts from (b) that do not have a *B. rapa* genome blast hit	3358	1675
f) Number of novel transcripts from (e) remaining after blasting to NCBI nr database	1218	
Final number of novel transcripts (d) and (f) combined	10,706	4052

### Reference-based assembly pipeline to detect novel transcripts

Because reference-based methods are highly sensitive for transcriptome reconstruction, for the current study we used a total of 157,164,008 reads from three lanes only (see *Materials and Methods*) to align to *B. rapa* genome using TopHat. Transcripts were then assembled from the mapped fragments using the Cufflinks assembler, producing a total of 76,640 transcripts. We next used Cuffcompare and found that 49.7% of all the transcripts matched exactly to the annotated exons downloaded from BRAD. The remaining 50.3% transcripts were classified into different classes as shown in Table 6. These results reflect the potential incompleteness or gaps of the current annotation of the *B. rapa* as well as the complex nature of transcription and RNA processing. We extracted a total of 6700 unknown intergenic transcripts (Class Code = “u”) as putatively novel transcripts because they occur in intergenic regions without any *B. rapa* annotation. To find out which of these transcripts corresponded to known genes from other organisms, we ran BLASTP against the nr database. Here, almost half of 6700 transcripts had some nr annotation (3275) while the rest did not (3425).

**Table 6:** The number of transcripts assembled with Cufflinks and the percentage they represent in the assembly after Cuffcompare analysis

Class Code	No. of Transcripts	%
=	38,126	49.75
C	14	0.018
E	3149	4.11
I	527	0.69
J	23,008	30.02
O	2596	3.39
P	1515	1.98
S	229	0.30
U	6708	8.75
X	768	1.00
**Total**	76,640	100.00

Class codes described by Cuffcompare: =, exactly equal to the reference annotation; c, contained in the reference annotation; e, possible pre-mRNA molecules; I, an exon falling into a intron of the reference; j, new isoforms; o, unknown generic overlap with reference; p, possible polymerase run-on fragment; u, unknown intergenic transcript.

### Meta-assembly

Finally, the novel transcripts identified from *de novo*–based assembly pipeline (14,758) and reference-based assembly pipeline (6700) were assembled together using CAP3 assembler. Further filtering to remove ambiguous ORF transcripts, very long transcripts and short isoforms resulted in final number of 3537 transcripts (see *Materials and Methods*). We also found that a total of 1197 transcripts remained unmapped to the genome and, among them, 780 transcripts have plant hits to the NCBI nr database. The remaining 417 transcripts have no hits in the NCBI database. These transcripts along with their gene descriptions have been saved to File S5 and File S6, respectively.

### Comparison of different assemblers

**Figure 3:**
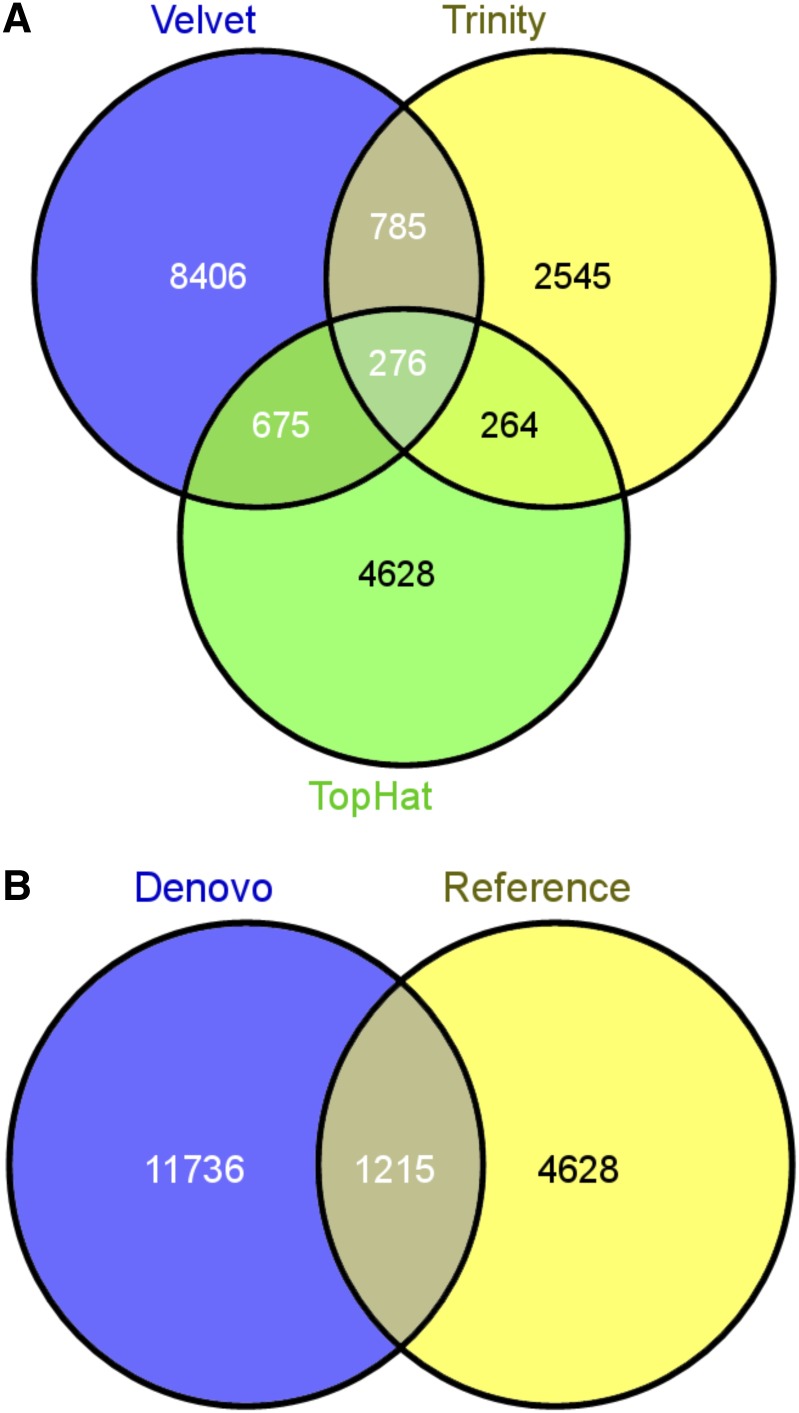
Venn diagrams showing unique and shared novel transcripts detected between (A) Velvet-Oases, Trinity, and TopHat-Cufflinks assemblers, (B) *de novo* (Velvet-Oases and Trinity), and reference-based (TopHat-Cufflinks) pipelines.

A comparison of the Velvet-Oases, Trinity, and TopHat-Cufflinks assemblers was performed using standard assembly statistics. The result showed that Velvet-Oases outperformed Trinity and TopHat-Cufflinks for all assembly parameters except for the percentage of annotated transcripts (Table 7). A higher percentage of annotated transcripts are generated by TopHat-Cufflinks even though fewer reads were used, consistent with the benefits of using the reference genome to anchor assemblies. We compared the novel transcripts of the three assemblers and found that there is only a marginal overlap, indicating that each transcriptome assembler has their own strengths (Figure 3A). The possible reasons for the marginal overlap are differences in the specifics of the assembly graphs, determination and weighting of graph edges, approaches to handle sequence errors, and approaches to handle diverse expression level.

**Table 7:** Comparison of assembly statistics from *de novo* (Velvet-Oases and Trinity) and reference (TopHat-Cufflinks) assemblers

	Velvet-Oases	Trinity	TopHat-Cufflinks
Total no. of reads	182,386,000	182,386,000	157,164,008
No. of initial transcripts	601,915	158,863	75,237
No. of transcripts after removing isoforms	43,816	39,084	53,632
Average size of transcript	1554	1112	1310
Maximum transcript length	26,060	22,887	16,681
Minimum transcript length	100	201	94
N50	2218	1863	1677
No. of transcripts in N50	134,411	28,901	18,762
% of transcripts annotated	67	84	87
No. of novel transcripts detected	14,540	5464	6700

The final output (novel transcripts) from the combined *de novo* assemblies (Velvet-Oases and Trinity) were next compared to the novel transcripts from reference-based assembly (TopHat-Cufflinks) and, as expected, the reference-based novel transcripts have a higher N50 than the *de novo*–based novel transcripts (Table 8). Interestingly, we found more unique novel transcripts in *de novo*–based assemblies compared with the reference-based assembly, probably due to the number of input reads (Figure 3B). Together, our assembly results show the importance of combining results from several different algorithms.

**Table 8:** Comparison of *de novo*–based and reference-based assembly methods on the final output (novel transcripts detected)

	*De novo*–Based	Reference-Based
No. of novel transcripts detected	14,758	6700
Maximum size of the transcript (bp)	7827	6897
N50 (bp)	640	1084

### Generating an updated annotation of the *B. rapa* genome

The PASA pipeline was used for updating the gene structure annotation of *B. rapa* genome (see *Materials and Methods*). A total of 23,754 genes were updated by this approach (Table 9), of which 293 genes were identified as "fused" (where two or more neighboring genes were fused as one gene).

**Table 9:** Comparison of original and updated *B. rapa* annotations using PASA

	First Update	Second Update	Third Update	Total
No. of gene models updated	23,132	597	25	23,754
No. of alternative splice isoforms	15,733	205	833	16,771
No. of annotated proteins changed	3505	132	18	3655

### Comprehensive *B. rapa* transcriptome and annotation

The 3537 novel transcripts along with their gene annotations were concatenated with 40,727 *B. rapa* transcripts (which includes both PASA updated and original transcripts) and their gene annotations. The final annotation set contained a total of 44,264 genes. We found that the final annotated *B. rapa* gene models were notably improved compared with older annotation based on visual inspection on IGV (File S7) and also in terms of mapping, for example, the percentage of RNA-Seq reads mapped was 2.5% higher in the updated annotation compared with older annotation. The updated gene annotation file (bed) and updated gene description file have been saved in File S8 and File S9, respectively.

### Validation of assembled novel transcripts

To test the authenticity of *de novo* and reference assembled novel transcripts, semi-quantitative RT-PCR was performed on 70 different novel transcripts. Agarose gel electrophoresis showed that 46 of the 70 primer pairs obtained a band of the expected size in at least one tissue type (Table 10). Out of the remaining 24 primer pairs that did not have bands, 14 of them had very low expression counts (0–5 mapped reads) in the tissues that were tested by RT-PCR, so they may be authentic but not expressed in the tissue available for testing. The remaining 10 with no bands may be due to failed PCR, bad primers, or poor assemblies because all of them have higher expression counts. Overall, deep RNA-Seq analysis was able to find novel transcripts and novel exons outside the current *B. rapa* annotation, with the majority being validated by RT-PCR.

**Table 10:** RT-PCR validations of assembled transcripts from different assembly methods

Assembly Type	Total No. of Genes Tested	No. of Genes Validated	Percentage of Validation
Velvet-Oases	20	13	65
Trinity	20	13	65
TopHat-Cufflinks “u” transcripts	20	14	70
TopHat-Cufflinks “o” transcripts	10	6	60

### Validation of novel and PASA updated transcript RNA-Seq structure and annotation

Our updated *B. rapa* transcriptome was generated from strain R500. It is important to ask if the differences between our R500 updated transcriptome and the original Chiifu annotation from BRAD are due to structural differences in the R500 and Chiifu transcriptomes, or if our updated R500 transcriptome represents a valid improvement for Chiifu. To do this, we used an *in silico* validation procedure, taking advantage of publicly available Chiifu RNA-Seq reads. We randomly chose 60 novel transcripts and 60 PASA updated transcripts and visualized the RNA-Seq coverage for R500 and Chiifu reads, along with the original and updated genome annotation in IGV (see *Materials and Methods*). There were a total of 12 transcripts where alternative splicing or low coverage precluded full classification; of the 108 transcripts that we could classify, the transcript structure of R500 and Chiifu was the same for 94% of them (Tables 11 and 12). The *de novo* transcripts matched the RNA-Seq data for both genotypes 70% of the time (Table 11). There were only three transcripts (6%) where the *de novo* annotation matched R500 but not Chiifu (Table 11). Focusing on the PASA updated annotations, 50 updated annotations (91%) matched both genotypes (Table 12). In contrast there were only two transcripts (4%) where the original annotation was better than the update for Chiifu (Table 12). In summary, the *de novo* and PASA updated transcripts represent a significant improvement for both the R500 and Chiifu genotypes. The genes used for *in silico* validation are given in Table S4 and Table S5, and IGV screen shots for both PASA and novel transcript annotations have been saved in File S7 and File S10.

**Table 11:** Summary of *in silico* validation of novel transcripts

		R500 and Chiifu Transcript Structure		
		R500 = Chiifu	R500 Different from Chiifu	Uncertain
**Comparison of RNA-Seq with *de novo* annotation**	Matches both	38		
	Matches R500		3	
	Matches Chiifu		1	
	Matches neither	11		
	Uncertain	1	2	4

Transcripts were classified as uncertain when alternative splicing or low coverage precluded definitive assignment.

**Table 12:** Summary of *in silico* validation of PASA updated transcript annotations

		R500 and Chiifu Transcript Structure	
		R500 = Chiifu	R500 Different from Chiifu
**Comparison of RNAseq with original and updated annotation**	Updated annotation correct for both	50	
	Annotation matches genotype		2
	Both annotations wrong	2	1
	Uncertain	4	1

Transcripts were classified as uncertain when alternative splicing or low coverage precluded definitive assignment.

### Functional annotation of revised *B. rapa* transcriptome

**Figure 4:**
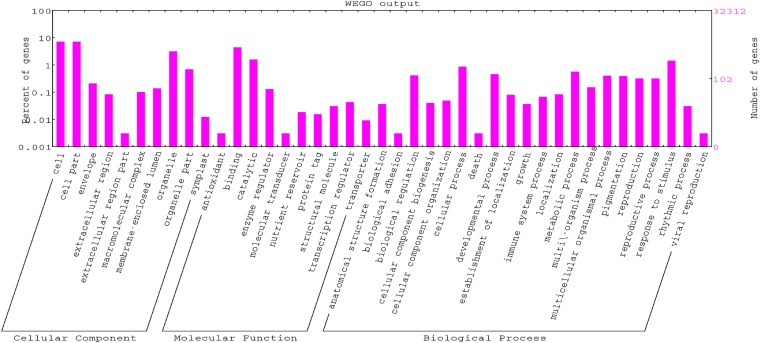
Histogram of level 2 GO term assignment of *B. rapa* re-annotated gene models. Results are summarized for three main GO categories: biological process (P), molecular function (F), and cellular component (C).

Functional annotation based on BLASTX and gene ontology allowed the classification of re-annotated transcripts into functional groups. The categorization of all BLASTX results indicate that *Arabidopsis thaliana*, *Vitis vinifera*, *Populus trichocarpa*, *Oryza sativa*, and *Ricinus communis* were the top five plant species in terms of number of hits to the revised transcriptome. The annotation step of Blast2GO assigned functions to 32,317 (73%) gene models. A total of 155,618 GO terms were obtained for a total of 44,239 re-annotated gene models. The distribution of the gene models in different GO categories is shown in Figure 4. Blast2GO can also be used to integrate other information such as metabolic pathways using KEGG annotation (Kanehisa and Goto 2000). We mapped all the predicted proteins to the reference canonical pathways in KEGG for functional categorization and annotation (see *Materials and Methods*). Of the 44,264 gene models, 13,203 had one or more KEGG annotations belonging to 321 different KEGG pathways. These GO and KEGG annotations will be helpful to researchers using the updated transcripts, for example, enabling categorization of transcriptional responses by the types of enriched GO or KEGG terms. The GO annotated table for *B. rapa* is saved in File S11.

## Conclusions

In this study, we have demonstrated that deep RNA-Seq of two genotypes using different tissues, growth conditions, and environments provides enough coverage for the detection of a large number of polymorphisms, discovery of unknown transcripts, and an update of existing annotation. Our results showed that no transcriptome assembler in either *de novo* assembly category or reference-based category is the best choice for transcriptome assembly and integrating assemblers from both the categories can offer more accurate assembly. We found that the sub-genome distribution can explain some, but not all, of the chromosomal variance in *KaKs*. Finally, even though there is divergence between R500 and Chiifu in terms of SNPs, we found that this divergence is not high enough to cause significant structural divergence of the transcriptome, as the updated annotation (both novel and PASA) provided a significant improvement to the existing annotation for both genotypes. The development of functional gene-based markers using RNA-Seq between contrasting genotypes in this study will help researchers using the parental strains and derived RILs for QTL and eQTL mapping. Ultimately, they could serve as excellent markers for marker-assisted selection in crop breeding.
